# Supplementary material for: The Systems Biology Research Tool: evolvable open-source software
Source: BMC Syst Biol. 2008 Jun 29;2:55. doi: 10.1186/1752-0509-2-55 (PMC2446383; doi:10.1186/1752-0509-2-55)
Supplement: Additional file 1 — SBRT Archive. An archive of the current version of the Systems Biology Research Tool. [file 1752-0509-2-55-S1.zip › sbrt-1.4.0/doc/developers_guide/api/sbrt/shell/text/math/BoundedIntervalFormatV1.html]

BoundedIntervalFormatV1


|  |  |  |
| --- | --- | --- |
| **Overview** \| **Package** \| **Class** \| **Use** \| **Tree** \| **Deprecated** \| **Index** \| **Help** | |  |
| PREV CLASS   **NEXT CLASS** | **FRAMES**    **NO FRAMES**     **All Classes** |  |
| SUMMARY: NESTED \| FIELD \| CONSTR \| METHOD | DETAIL: FIELD \| CONSTR \| METHOD |  |


---


## sbrt.shell.text.math Class BoundedIntervalFormatV1

```
java.lang.Object
  sbrt.shell.text.math.BoundedIntervalFormatV1
```

**All Implemented Interfaces:**: Format, Formatter<Interval>, IntervalFormat, Parser<Interval>, SimpleFormat<Interval>

---

``` public final class BoundedIntervalFormatV1 extends java.lang.Object implements IntervalFormat ```

This class is a concrete implementation of `IntervalFormat`. The
string representation of intervals used by this class is:
`[LowerBound; UpperBound]`. The bounds must be finite.

**Author:**
:   This class was written and documented by Jeremiah Wright while in the
    Wagner lab.

---

**Constructor Summary**

`BoundedIntervalFormatV1()`
  
          Constructs a new interval format.


| **Method Summary** | |
| --- | --- |
| `java.lang.String` | `format(Interval interval)`             Returns a formatted string representation of the provided interval. |
| `DoubleFormatV1` | `getDoubleFormat()`             Returns the double format used for bounds. |
| `IntervalFormatV1` | `getIntervalFormat()`             Returns the interval format used by this format. |
| `Interval` | `parse(java.lang.String interval)`             Parses the provided string and returns its corresponding interval. |

**Methods inherited from class java.lang.Object**

`clone, equals, finalize, getClass, hashCode, notify, notifyAll, toString, wait, wait, wait`

**Constructor Detail**

### BoundedIntervalFormatV1

```
public BoundedIntervalFormatV1()
```

:   Constructs a new interval format.


**Method Detail**

### getIntervalFormat

```
public IntervalFormatV1 getIntervalFormat()
```

:   Returns the interval format used by this format.

    :   **Returns:**: the interval format used by this format.

---


### getDoubleFormat

```
public DoubleFormatV1 getDoubleFormat()
```

:   Returns the double format used for bounds.

    :   **Returns:**: the double format used for bounds.

---


### format

```
public java.lang.String format(Interval interval)
```

:   Returns a formatted string representation of the provided interval.

    :   **Specified by:**: `format` in interface `Formatter<Interval>`
    :   **Parameters:**: `interval` - the interval to be formatted. **Returns:**: a formatted string representation of the provided interval. **Throws:**: `java.lang.IllegalArgumentException` - if the provided interval is unbounded.

---


### parse

```
public Interval parse(java.lang.String interval)
```

:   Parses the provided string and returns its corresponding interval.

    :   **Specified by:**: `parse` in interface `Parser<Interval>`
    :   **Parameters:**: `interval` - the string to be parsed. **Returns:**: the interval corresponding to the provided string. **Throws:**: `FormatException` - if the interval cannot be parsed, or if a bound of the parsed interval is infinite.


---


|  |  |  |
| --- | --- | --- |
| **Overview** \| **Package** \| **Class** \| **Use** \| **Tree** \| **Deprecated** \| **Index** \| **Help** | |  |
| PREV CLASS   **NEXT CLASS** | **FRAMES**    **NO FRAMES**     **All Classes** |  |
| SUMMARY: NESTED \| FIELD \| CONSTR \| METHOD | DETAIL: FIELD \| CONSTR \| METHOD |  |


---
